# Andrew Mangham 2020: *The Science of Starving in Victorian Literature, Medicine, and Political Economy* und Elizabeth A. Williams 2020: *Appetite and Its Discontents. Science, Medicine, and the Urge to Eat, 1750–1950*

**DOI:** 10.1007/s00048-021-00312-9

**Published:** 2021-09-13

**Authors:** Nina Mackert

**Affiliations:** Universität Leipzig, Leipzig, Deutschland

**Andrew Mangham 2020:** ***The Science of Starving in Victorian Literature, Medicine, and Political Economy.*** Oxford: Oxford University Press, geb., 240 S., 55 £, ISBN: 978-0-1988-5003-8.

**Elizabeth A. Williams 2020:** ***Appetite and Its Discontents. Science, Medicine, and the Urge to Eat, 1750–1950.*** Chicago: University of Chicago Press, brosch., 416 S., 10 Abb., 35 $, ISBN: 978-0-2266-9304-0.

Studien zur Wissenschaftsgeschichte des Essens haben seit gut einer Dekade Konjunktur, insbesondere im Zusammenhang mit einer intensiven Problematisierung von Essen und Ernährung in unserer Gegenwartsgesellschaft. Zwei neue Monografien widmen sich nun der Wissenschaftsgeschichte von Hunger beziehungsweise Appetit. Bei aller Unterschiedlichkeit teilen Andrew Manghams *The Science of Starving* und Elizabeth A. Williams’ *Appetite and its Discontents* nicht nur ihre Betonung der Schwierigkeit, Hunger und Appetit zu erfassen. Sie kreisen auch beide um die für gegenwärtige Auseinandersetzungen um „richtiges“ Essen zentrale Frage nach der individuellen Verantwortung für die eigene Gesundheit.

Manghams Studie nimmt den Hunger ins Visier. Er beschäftigt sich mit dem Einfluss physiologischen und medizinischen Wissens auf die Sozialromane von Charles Kingsley, Elizabeth Gaskell und Charles Dickens. Mangham erweitert die gängige Diagnose, dass Wissenschaft im 19. Jahrhundert zunehmend als Ressource für das Ordnen von Gesellschaft begriffen wurde, indem er zeigt, wie Sozialromane sprachliche und epistemologische Register der „science of starving“ (3) nutzten, um Hunger und Armut zu kritisieren und als unnatürlich darzustellen. Es sei dieser „rich and dynamic interchange […] between the period’s fiction and biological science“ (14), der die bereits von James Vernon diagnostizierte „humanitäre Entdeckung“ des Hungers seit den 1840er-Jahren mit hervorgebracht habe.

Zwar behandelt Mangham nur eine Richtung dieses Austauschs, dies tut er aber sehr gewinnbringend. Er argumentiert, dass sich Sozialromane sowohl die materialistische Sprache aus Physiologie und Biologie als auch deren experimentellen Blick auf Hunger als schwer klassifizierbares Problem aneigneten. Damit forderten sie vorherrschende nationalökonomische Konzepte heraus, die Hungersnöte mit Thomas Malthus als notwendiges Korrektiv von Überbevölkerung und individuellen Verfehlungen konturierten. Erschien hier Hunger als Resultat der Verletzung einer göttlichen Ordnung und mithin als natürlich oder gar heilsam, setzte der Fokus auf physische Prozesse des Hungerns und des Verfalls ein „alternative narrative of hunger as unnecessary, unjust, and unnatural“ (18) dagegen.

Nach einem Kapitel, das sich diesen konzeptionellen Differenzen widmet, räumt Mangham jeweils ein Kapitel der detailreichen Analyse der Werke von Kingsley, Gaskell und Dickens ein. Kingsleys Arbeiten zeigen die Spannung zwischen materialistischen und providentialistischen Ideen besonders deutlich, ohne sich eindeutig einem der beiden Pole zuordnen zu lassen. Gleichzeitig seien diese aber in ihrer Darstellung von Hunger als komplexem und schwer zu erfassendem Phänomen durch den „methodological scepticism“ (71) von Biologie und Physiologie geprägt, den Mangham als charakteristisch für den „self-reflexive realism of the social problem novel“ (12) ansieht.

Auch bei Gaskell zeigt Mangham, wie sie in ihren Arbeiten der abstrakten, „hygienischen“ Sprache der politischen Ökonomie eine konfrontative, detailreiche Beschreibung der physischen Realität des Hungerns entgegensetzt. Insbesondere Gaskells Werke zeigen zudem, dass es bei der Kritik der Sozialromane an Hunger als göttlicher Fügung nicht schlicht um eine allein rationale Kritik ging, sondern vielmehr darum, spirituell-moralische Antworten zu provozieren.

In der Relektüre von Dickens zeigt Mangham ebenfalls, dass dessen polemische Kritik an Ignoranz und Dogmatismus der politischen Ökonomen – wohl am bekanntesten verkörpert in der Figur des Ebenezer Scrooge – über eine Aneignung physiologischer Sprache und Konzepte funktionierte. In diesem Kapitel wird ein Aspekt besonders explizit, den Mangham im Schlusskapitel wieder aufgreift: die Frage von Verantwortung. Dickens’ Werke forderten ein Verständnis von Hunger als Resultat individueller Verantwortungslosigkeit heraus, indem sie das Hungern als unnatürlich und zu komplex für eine simple Darstellung in den Statistiken der Politischen Ökonomie konturierten.

Wird bei Mangham immer wieder deutlich, dass Hunger ein „paradigm of unstable knowledge“ (128) ist und sich einfachen Interpretationen entzieht, steht eine solche Unbestimmtheit im Zentrum von Williams’ Wissenschaftsgeschichte des Appetits. Hier geht es darum, zu zeigen, wie Naturwissenschaftler und Ärzte (die behandelten Forscher sind fast ausnahmslos männlich) über zwei Jahrhunderte hinweg versuchten, den Appetit einzukreisen, ihn in Körper und/oder Geist zu verorten, in seinen „gesunden“, „normalen“ oder „pathologischen“ Dimensionen zu erfassen und zu erklären. Kurz, es geht Williams darum, wie „appetite – once a simple reality of daily life – became an ‚object‘ to be defined and, ultimately, managed by scientists and pyhsicians“ (5). In diesem „ultimately managed“ deutet sich Williams’ wichtigstes Argument an. Die „zentrale Dynamik“ (15) der naturwissenschaftlichen und medizinischen Beschäftigung mit dem Appetit zwischen 1750 und 1950 (und in den Dekaden darauf, wie sie im Schluss skizziert) war die Tendenz zur „Homogenisierung“: „a drive toward uniformity justified by apparently unchallengeable values of health and longevity“ (15).

So sehr Williams diese Tendenz zur Universalisierung des Appetits in Einleitung und Schluss betont: Ihre kenntnisreichen, quellennahen und -gesättigten Ausführungen dazwischen zeichnen ein viel uneinheitlicheres und widersprüchlicheres Bild der Appetitforschung. Die Disziplinen, die Williams auch immer wieder in ihrer Herausbildung und gegenseitigen Abgrenzung untersucht, sind Physiologie, Naturgeschichte und Medizin, ab dem späten 19. Jahrhundert dann auch Psychologie, Anthropologie und Verhaltensforschung. Dabei konzentriert sie sich vor 1900 auf Europa, danach auf die USA und den transatlantischen Raum. Gegliedert hat sie ihre Studie in vier Abschnitte, die jeweils fünf Dekaden umfassen und mit disziplinär getrennten Unterkapiteln als grobe Einteilungen von Verschiebungen in der Erforschung des Appetits fungieren – Verschiebungen, die jedoch nicht ganz klar entlang der Einteilung der Abschnitte auszumachen sind.

Deutlich hilfreicher, um die Beschäftigung mit dem Appetit in Williams’ Darstellung zu erfassen, sind daher drei eng miteinander verknüpfte Themen, die sich in der *longue durée* immer wieder zeigen. Erstens macht Williams deutlich, wie der Appetit sich dem beobachtenden und vermessenden Zugriff von Medizin und Naturwissenschaften stets widersetzte, weil er sich nicht quantifizieren, eindeutig verorten oder klar vom Hunger abgrenzen ließ. Sie zeichnet nach, wie Vertreter verschiedener Disziplinen immer wieder darum bemüht waren, den Appetit in Körper oder Geist zu lokalisieren; so etwa mechanistische Physiologen im 18. Jahrhundert, die Appetit entgegen vitalistischen Auffassungen als Empfindung konzipierten, die ihren Ursprung im Nervensystem habe. Mit der zunehmenden Hinwendung zu Experiment und Labor seit dem 19. Jahrhundert sei der Appetit zugunsten von beobachtbaren Aspekten von Essen und Verdauung leichter aus dem Blickfeld der Disziplinen geraten. Gleichzeitig regte diese Unklarheit immer wieder Forderungen an, den Appetit „ganzheitlich“ und als Kennzeichen „psychosomatischer“ Vorgänge zu betrachten.

Zweitens beschäftigten sich die Wissenschaften vom Appetit immer wieder mit der Frage, ob dieser individuell sei oder sich überindividuelle Regelmäßigkeiten feststellen ließen – eine Frage, die überaus wichtig war für die Entwicklung von allgemeine Gültigkeit beanspruchenden Ernährungsempfehlungen. Dabei wird deutlich, dass die Tendenz zur „Standardisierung“ des Appetits, die gerade mit der Durchsetzung experimenteller Methoden an Fahrt aufnahm, auch immer wieder herausgefordert wurde. Dies geschah zum Beispiel durch ärztliche „bedside observations“, die bis ins 20. Jahrhundert auf individuelle Besonderheiten von Appetit und Essverhalten konzentriert waren.

Drittens zeigt Williams Auseinandersetzungen um die Frage auf, ob Appetit eine instinktive und mithin verlässliche Orientierungshilfe bei der Auswahl des „richtigen“ Essens war oder aber erlernt und stets sorgfältig gemanagt werden musste – und damit für die moralische Bewertung individueller Selbstführung kennzeichnend sein konnte. Diese differente Konzeption von Appetit war etwa entscheidend für die Frage, ob die seit dem 18. Jahrhundert verstärkt diskutierte „corpulence“ und später „obesity“ als Folge pathologischer Materie, wie noch bis zum späteren 19. Jahrhundert vorherrschend, oder als Resultat mangelnder Selbstkontrolle gedacht wurde.

Trotz dieser folgenschweren Implikationen wissenschaftlicher Konzeptionen des Appetits bleibt Williams, im Gegensatz zu Mangham, hinsichtlich der Frage nach der gesellschaftlichen Produktivität des wissenschaftlichen Wissens bedauerlicherweise eher stumm. Hier und da scheinen gesellschaftliche Felder auf, in denen das Appetitwissen wirksam wurde – etwa in den (leider äußerst knappen) Abhandlungen zu geschlechtsspezifischen Differenzierungen von Appetit. Zwar liefert Williams in ihren sehr hilfreichen Einleitungen zu den vier Blöcken etwas kultur- und gesellschaftsgeschichtlichen Kontext, insgesamt aber will das Buch explizit in der innerwissenschaftlichen Auseinandersetzung um Appetit bleiben. Die untersuchten Wissenschaftler, so Williams, begriffen Appetit als „object isolated from ‚real-world‘ contexts of food and eating“ (6) und entwickelten ihre Argumente vor allem in der Auseinandersetzung mit innerwissenschaftlichen Debatten und nicht als Antwort auf gesellschaftliche Fragen. Dies überzeugt angesichts der enormen gesellschaftspolitischen Bedeutung von Auseinandersetzungen um Essen, Körper und individuelle Verantwortung spätestens seit dem 19. Jahrhundert, die Williams selbst in ihrer Einleitung und im Schlusskapitel aufruft, jedoch nicht ganz.

Nichtsdestotrotz ist Williams’ Studie eine lehrreiche und anregende Fundgrube von Appetitwissen über mehr als zwei Jahrhunderte. Gerade in der Kombination mit Manghams Buch erinnert sie daran, wie fruchtbar die Historisierung von Essen und Ernährung vor dem Hintergrund gesellschaftlicher Auseinandersetzungen um Verantwortung ist.

